# Gene Expression Analyses in Non Muscle Invasive Bladder Cancer Reveals a Role for Alternative Splicing and Tp53 Status

**DOI:** 10.1038/s41598-019-46652-4

**Published:** 2019-07-17

**Authors:** Marta Dueñas, Andrés Pérez-Figueroa, Carla Oliveira, Cristian Suárez-Cabrera, Abel Sousa, Patricia Oliveira, Felipe Villacampa, Jesús M. Paramio, Mónica Martínez-Fernández

**Affiliations:** 1 Molecular Oncology Unit, CIEMAT, Avda Complutense 40, 28040 Madrid, Spain; 2 Biomedical Research Institute, Hospital Universitario 12 de Octubre, Avda Córdoba s/n, 28041 Madrid, Spain; 3 CIBERONC, Biomedical Research Networking Centers, Madrid, Spain; 4 Phylogenomics Lab. Department of Biochemistry, Genetics an Immunology & Biomedical Research Center (CINBIO), University of Vigo, 36310 Vigo, Spain; 5 Expression Regulation in Cancer Lab, Universidade do Porto, i3s & IPATIMUP. Rua Alfredo Allen, 208, 4200-135 Porto, Portugal; 6 Present Address: Urology Department, Clinica Universidad de Navarra, Madrid, Spain; 7 Present Address: Genomes and Disease Lab. Center for Molecular Medicine and Chronic Diseases Research (CIMUS), Universidade de Santiago de Compostela (USC), Avda de Barcelona, 31, 15706 Santiago de Compostela, Spain

**Keywords:** Bladder cancer, Bladder

## Abstract

Non-muscle invasive bladder cancer (NMIBC) represents a crucial problem for the national health care systems due to its high rates of recurrence and the consequent need of frequent follow-ups. Here, gene expression analyses in patients diagnosed as NMIBC were performed to determine those molecular pathways involved in tumor initiation, finding that both MYC and E2F are up regulated and helps to tumor initiation and progression. Our results also support an important involvement of alternative splicing events, modifying key pathways to favour bladder tumor evolution. Finally, since MDM2 showed differential exon usage, mutations in TP53 and its protein expression have been also studied in the same patients. Our data support that recurrence is epigenetically mediated and favoured by an increase protein expression of TP53, which appears more frequently mutated in advanced stages and grades, being associated to a worse prognosis. Therefore, TP53 mutational status could be used as a potential biomarker in the first stages of NMIBC to predict recurrence and prognosis.

## Introduction

Bladder cancer (BC) is the tenth most frequently diagnosed tumor worldwide and entails the highest cost per patient among all types of cancers^1^. At diagnosis, tumor appears frequently as non-muscle invasive bladder cancer (70% NMIBC; Ta-T1 stages), while a lower percentage is already invasive into the bladder muscle layers (30% MIBC; T2-T4 stages). In these last cases, the gold standard for treatment is a radical cystectomy preceded, when possible, by cisplatin-based chemotherapy with the aim of preventing the frequent metastases. In the case of NMIBC, it has a priory higher life expectancy, and its treatment consists of a transurethral resection (TUR), sometimes followed by bladder instillations of BCG or mitomycin. In spite of this invasive treatment, NMIBC has extremely high rates of recurrences (70–80%), and some recurrences can even appear with an advanced tumor stage and/or grade (30%) with the associated worse outcome. Currently, the only procedure to monitor these patients and to prevent recurrence is a frequent cystoscopy. Unfortunately, this impairs patients’ life quality and has high costs for the national health systems. Although important efforts have been recently done^2–5^, there is still an urgent need for a better understanding of the molecular mechanisms behind the high rates of recurrence and for a predictable biomarker.

Gene expression analyses in NMIBC patients pointed towards a role of alternative splicing (AS) for tumor initiation. AS events in the human genome provides the capability to diversify the proteome through the alternative combination of exons from a single gene to form different mature mRNAs and protein products. Using different transcriptomic technologies, it is possible to study gene expression at the exon resolution, allowing the detection of AS events that could be altering cancer key pathways. In fact, recent studies have shown that AS is frequently altered in several tumor types and that the new aberrant isoforms can act as cancer drivers, affecting to fundamental oncogenic processes such as angiogenesis, proliferation, immune escape, apoptosis, or metastasis^6,7^. Although different associations between particular AS events and patient prognosis have been reported in several tumor types^8,9^, little is still known in the case of BC^10–12^. Here, we have carried out the first comprehensive AS related study in a cohort of 82 NMIBC patients (Fig. S1A). Our results support a key role of AS able of modulating expression of crucial pathways, favouring tumor progression. In addition, following the results found, single nucleotide variations in *TP53* gene were analysed using Next Generation Sequencing. This is especially interesting since *TP53* has been frequently detected as mutated and related to chemo-resistance in MIBC^13–16^, but its role and status in NMIBC is still to be clarified. Our results support that TP53 mutations and expression can be used to determine patients’ prognosis.

## Results

### Alternative splicing events in BC

**Figure 1:**
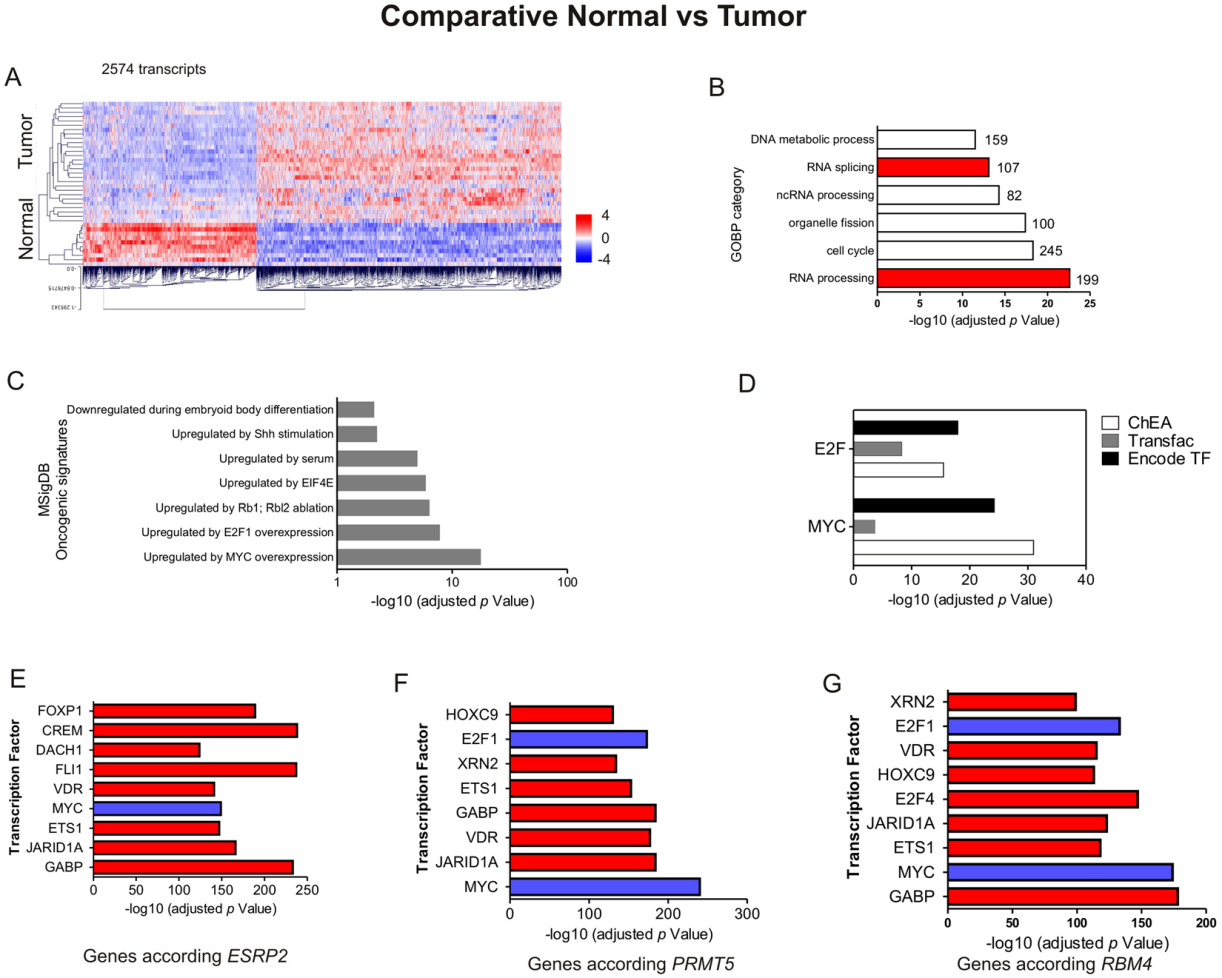
(**A**) Differential gene expression between normal and bladder cancer samples using Affymetrix HuGene-1_0-st-v1 arrays. (**B**) Gene Ontology Biological Processes (GOBP) analyses of the transcripts showing statistically significant differences. (**C**) Genesets often de-regulated in cancer that overlaps with the genesets showing differences between normal and tumor samples using the Molecular Signatures Database (MSigDB) from GSEA software. (**D**) Those de-regulated genes share binding motifs for *MYC* and *E2Fs* transcription factors, according with ChEA, Transfac, and Encode Transcription Factor databases. (**E**) Genes whose expression is correlated with the expression of the *ESRP2* splicing factor. (**F**) Genes whose expression is correlated with the expression of the *PRMT5* splicing factor. (**G**) Genes whose expression is correlated with the expression of the *RBM4* splicing factor.

Affymetrix HuGene-1_0-st-v1 arrays were used to interrogate 10 normal and 28 bladder cancer samples (GSE38264^4^). As shown in Fig. 1A, differential gene expression in 2574 transcripts could clearly differentiate normal versus tumor samples. Gene Ontology Biological Processes (GOBP) analyses on these transcripts revealed, among other processes, an important role of AS events and RNA processing (Fig. 1B). Expression of genes differentially expressed was compared to those gene-sets included in the Oncogenic Molecular Signatures Database (MSigDB) frequently deregulated in cancer. We found that genes with differential expression matched with signatures of key oncogenic pathways such as those upregulated by Shh stimulation, MYC overexpression, E2F1 upregulation, or by Rb1-Rbl2 ablation (Fig. 1C). In agreement, using ChEA, Transfac, and Encode Transcription Factor databases, we also observed that these deregulated genes presented binding motifs for both *MYC* and *E2Fs* transcription factors (Fig. 1D). To further understand the role of AS, we decided to test which genes showed a statistical expression correlation with different splicing factors expression included in the array, such as *ESRP2*, *PRMT5*, and *RBM4*. Using a PTM analysis, we found a high statistically significant correlation between the expression of these splicing genes and important cancer-related transcription factors (Fig. 1E–G), including genes involved in cancer (*ETS1*, *XRN2*, *VDR*), in epigenetic mechanisms (*JARID1A*, *HOXC9*), and once again *MYC* and *E2F* transcription factors.

Since these results supported such involvement of *MYC* and *E2F* activators, we decided to measure their expression in a more extensive dataset including 77 bladder tumours and their corresponding normal samples (Fig. S1A). We could confirm that the activator members of *E2F* family, namely *E2F1* and *E2F2*, showed also an up-regulation in tumor samples compared to normal samples (Fig. S1B), confirmed by the previously described overexpression of *E2F3a* in this NMIBC dataset^4^. In the case of *cMYC*, a higher statistically significant expression was also detected in the bladder tumors in comparison with their corresponding healthy bladder tissue (Supl Fig. 1). These results support that both *cMYC* and *E2Fs* up-regulation is characteristic of NMIBC.

### Differential exon expression between normal and non-invasive tumor bladder samples

**Figure 2:**
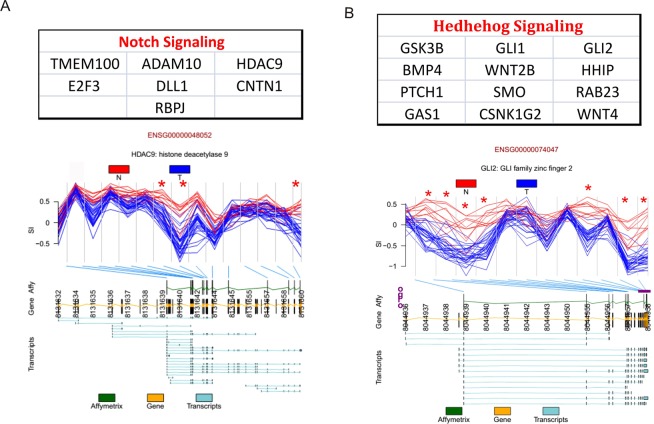
Splicing index (SI) analyses using Affymetrix HuGene-1_0-st-v1 comparing each tumor bladder cancer sample with its corresponding normal bladder sample. (**A**) Genes belonging to Notch pathway showing alternative splicing events. HDAC9 is shown as example. Red lines represent the SI from recurrence samples and blue from tumor samples without recurrence development. Green boxes represent where the array probes are located. Yellow boxes represent exon genes. Horizontal blue lines represent the different transcripts described according Ensemble database. Only some of the exons (yellow boxes) have representation in the array (green boxes). (**B**) GOBP analyses showed also an important number of Hedgehog pathways genes with AS. In this case GLI2 gene is represented as example. Red lines represent the SI from recurrence samples and blue from tumor samples without recurrence development. Green boxes represent where the array probes are located. Yellow boxes represent exon genes. Horizontal blue lines represent the different transcripts described according Ensemble database. Only some of the exons (yellow boxes) have representation in the array (green boxes).

**Figure 3:**
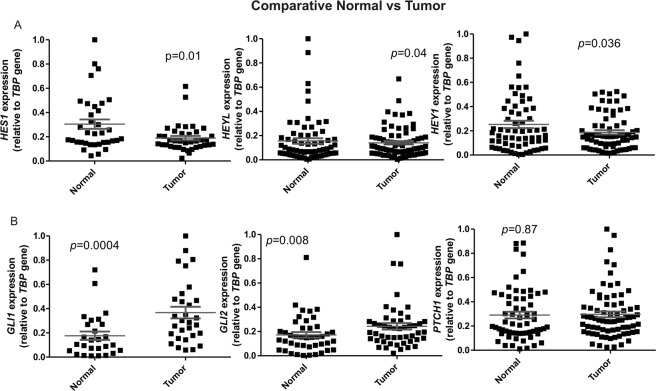
RT-qPCR analyses using *TBP* as gene normalizer. (**A**) Notch read-out (*HES1*, *HEYL*, and *HEY1*) gene expressions were measured, finding decreased expressions in tumor samples compared with paired normal samples. (**B**) Hedgehog read-out (*GLI1*, *GLI2*, and *PTCH1*) gene expressions were measured, finding increased expression in tumor samples.

Since Affymetrix HuGene-1_0-st-v1 arrays include several probes at exon level, we could develop a bioinformatic pipeline to study the differences in exon usage based on the splicing index calculation. The splicing index (SI) is the log ratio of the exon intensities after normalization to the gene intensity, and it was calculated for each exon for all the samples analyzed by Affymetrix HuGene-1_0-st-v1. After data processing, a total of 174616 exons were detected. Among them, 3443 showed statistically significant differences in their expression between normal and tumor samples (Benjamini-Hochberg multiple test correction *p* < 0.001), belonging to 1747 genes. GOBP analyses revealed different pathways altered, including several genes belonging to Notch and Hedgehog signalling (Fig. 2B,C), previously reported as key for BC progression^17,18^. As a whole, most of the exons showing differential expression appeared less expressed in tumor bladder samples, suggesting a tendency of loss of exons that may lead to new shorter isoforms, more efficient to favour tumor progression. Since studying each new isoform expression and activity will be out of the current scope, and to further better understand how these AS events along different genes in these pathways were affecting the global Notch and Hedgehog activity levels, we decided to measure the expression of their corresponding readouts by RT-qPCR in the wider dataset (Fig. S1A). We could confirm that *HES1*, *HEYL* and *HEY1* showed statistically significant decreased expression in bladder tumor compared with normal tissue (Fig. 3A), supporting that the alternative isoforms collaborate with the previously described decreased Notch activity in BC^17^. In the case of Hedgehog, its main read-out genes are *PTCH1* together with *GLI1* and *GLI2*. Both *GLI1* and *GLI2* showed a statistically significant higher expression in tumor samples compared to normal bladder, while *PTCH1* did not show statistically significant differences (Fig. 3B). These results support that the AS events along different genes in the pathway help to the previously reported activation of Hedgehog activity in tumor samples, favouring progression of BC^18^.

### Differential exon expression between recurrent and non-recurrent bladder samples

**Figure 4:**
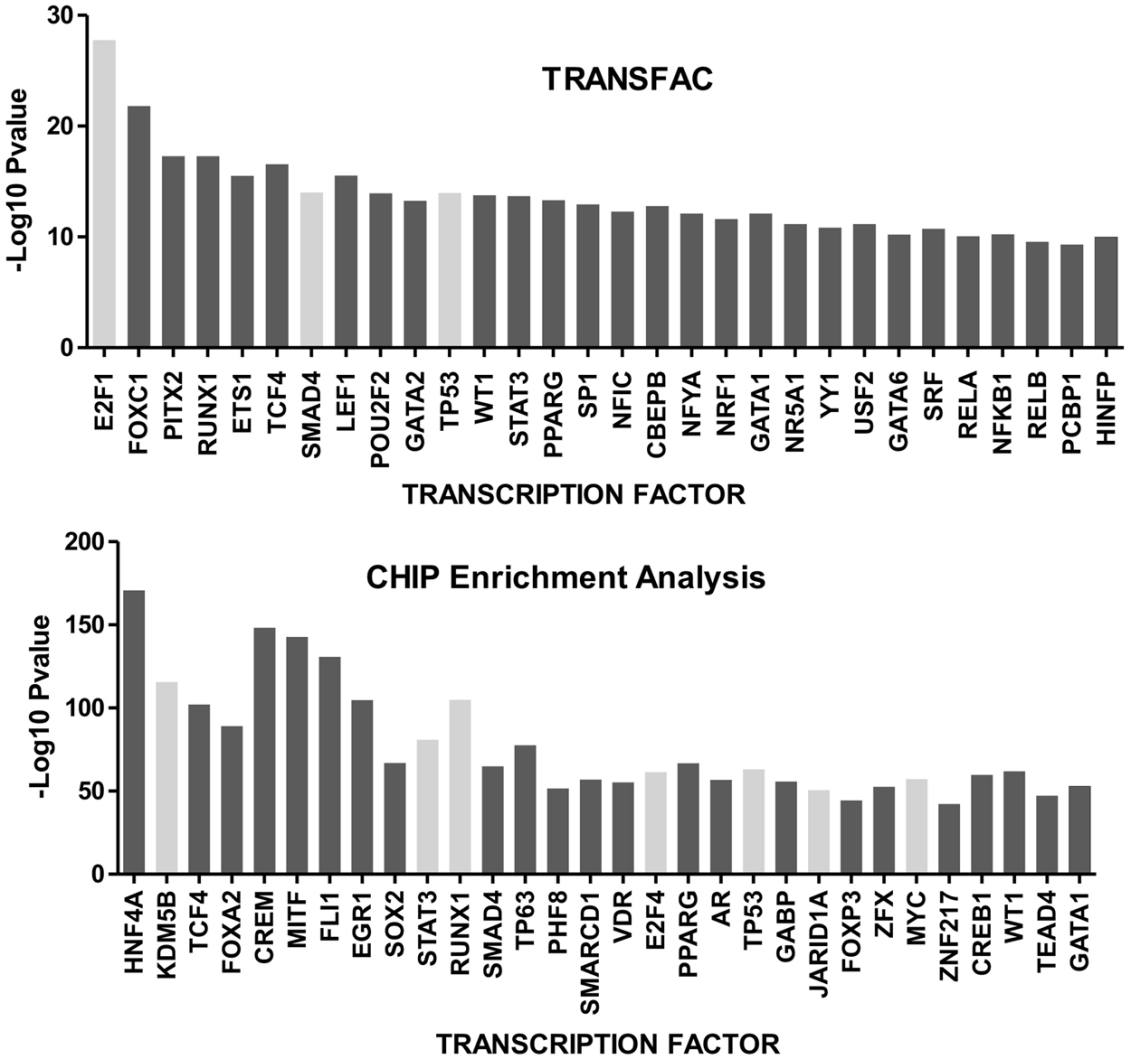
Splicing index (SI) analyses using Affymetrix HuGene-1_0-st-v1 comparing tumor bladder cancer samples with and without recurrences. The deregulated genes showed bindings for important transcription factor according TRANSFAC database and Chip Enrichment Analyses (ChEA).

Splicing Index (SI) was also calculated in a comparison between patient’s tumours suffering recurrence versus tumours without recurrence. In this case, 173812 exons could be evaluated, finding 2121 with statistically significant differences in expression (adjusted p-value < 0.01), belonging to 1761 genes. Gene Ontology (GOBP) analyses showed that most of these genes were involved in regulation of gene expression and epigenetic mechanisms (ie. gene silencing by miRNA, RNA processing, chromatin modification). Gene Set Enrichment Analyses (GSEA), using TRANSFAC database and Chip Enrichment Analyses (ChEA), showed that those genes with AS presented binding sites for key cancer-related transcription factors (*SMAD4*, *STAT3*, *RUNX1*, *TP53*,…), epigenetic factors (*JARID1A*, *KDM5B*), and again *E2Fs*, and *MYC* (Fig. 4). Therefore epigenetic factors and *E2F* and *MYC* are clearly involved not only in the tumor initiation but also in the recurrence development.

### Alternative splicing of MDM2 and TP53 expression in NMIBC

**Figure 5:**
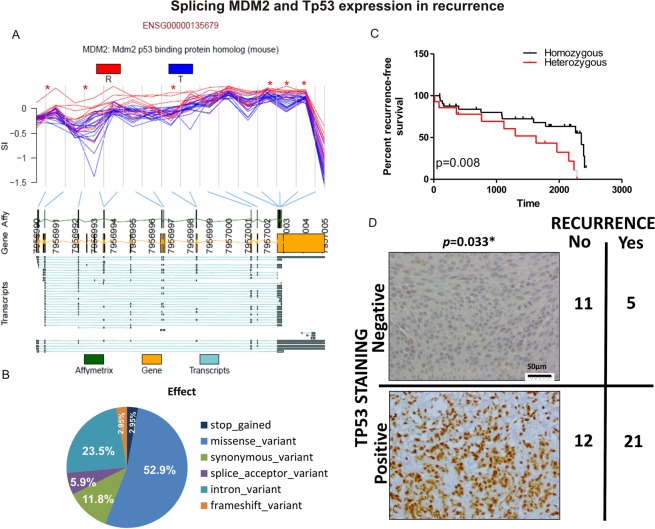
(**A**) *MDM2* gene showed exons differentially expressed between patients’ samples with (red) and without (blue) recurrence. Red lines represent the SI from recurrence samples and blue from tumor samples without recurrence development. Green boxes represent where the array probes are located. Yellow boxes represent exon genes. Horizontal blue lines represent the different transcripts described according Ensemble database. (**B**) Classification and percentage of the SNVs detected for *TP53* is patients’ samples. (**C**) Kaplan-Meier curves representing the recurrence-free survival differences between homozygous and heterozygous patients. Heterozygous patients showed statistically worse prognosis. (**D**) Example of a negative (up) and a positive (down) protein TP53 staining. The number of patients in each category in the contingency analysis was included and *p*-value was obtained by Fisher exact test.

When comparing samples from patients with and without recurrence, *MDM2* showed several exons with statistically significant differences (*p* = 0.0004), showing a higher expression of the first and last exons in the samples from patients developing recurrence (Fig. 5A). This result could indicate that there is a higher proportion of shorter *MDM2* alternative isoforms, described previously as unable to repress TP53, in recurrent tumours expression. This found, together with the previous result where genes with AS in recurrent samples showed binding sites for *TP53*, prompted us to check the expression of TP53, to confirm if it could be related to recurrence development. Then, its protein expression was evaluated in a Tissue MicroArray (TMA) containing the whole patients’ cohort (Fig. S1A). In this way, we detected tumours with positive and with negative staining, as shown in Fig. 5D. Interestingly, we could confirm that those patients with positive stained tumours (high expression of TP53) presented a higher likelihood of suffering recurrence (*p* = 0.033*), supporting that TP53 expression could be a good predictable biomarker for recurrence. Using the RNAseq data from Hedegaard *et al*.’s study, where 476 NMIBC are included^19^, we could confirm that TP53 appeared more expressed in more advanced stages (Ta vs T1, *p* = 0.041), showing the same tendency for high-grade tumours (*p* = 0.059), and for MIBC tumours, although not reaching the statistical significance probably due to the few invasive samples included (16 T2-4 vs 457 Ta-T1).

Based on these results, we decided to examine if the differences in TP53 expression could be also caused by DNA mutations. NGS-based analysis of *TP53* mutations was carried out using the TP53 MASTR from Multiplicon for those patients of the same dataset with DNA available (n = 61, Fig. S1A), targeting SNVs and small indels in the coding region of *TP53*. Of the 61 NMIBC patient samples, 40.4% presented some SNVs, finding a total of 34 different SNVs in the whole dataset. Regarding the type effect of these SNVs, 52.9% were missense variant, 23.5% intron variants, 11.8% synonymous variant, 5.9% splice acceptor variant, 2.95% frameshift variant, and 2.95% stop gained variants (Fig. 5B). The SNV presence was checked to find possible association with the patients’ clinic-pathological characteristics. Thus, we found statistically significant associations with the tumor stage (*p* = 0.017*), the tumor grade (*p* = 0.026*), and close to the statistical significance in the case of disease specific survival (dss) (*p* = 0.056). These differences were even more supported when only those SNVs in coding sequences were considered (29.8% of patients), finding statistically significant associations with tumor stage (*p* = 0.007**), tumor grade (*p* = 0.007**), and disease specific survival (*p* = 0.041*).

In addition, two population variants were found: a missense variant (rs1042522) in codon 72, and one intron variant (rs146534833). In the case of the intron, the variation consisted of an insertion variation that appeared in heterozygosis in 73.8% of the cases, in homozygosis in 3.3%, and absent in 22.9% of the patients. In the case of the variant in the codon 72, it is a common polymorphism, encoding either proline (cCc) or arginine (cGc). In our dataset, the allelic frequencies for the homozygous (C/C) were from 0.998 to 0.913 (mean = 0.992), while in the case of heterozygous (G/C) were from 0.291 to 0.845 (mean = 0.598). Most of the cases were homozygous for proline triplet (cCc: 64.7%). Remarkably, when they were compared to those heterozygous proline/arginine (cGc: 29.4%), homozygous displayed a better free recurrence survival being statistically significant (Fig. 5C).

## Discussion

Around 70–80% Non-Muscle Invasive Bladder Cancer (NMIBC) patients will suffer tumor recurrence after first surgical treatment. Therefore, it represents an important challenge for national health systems due to the consequent associated high costs for the treatment and the required continuous follow-up. In the present work, we found a differential gene expression pattern between tumor and normal bladder cancer samples. Expression analyses revealed a clear up-regulation of *c-MYC* and *E2F* transcription factor in tumor samples compared to their corresponding normal tissues showing also a correlation with splicing factors.

Regarding E2F activation in BC, *E2F3a* had already been earlier claimed as BC oncogene^20,21^. Previous data^4^ in NMIBC already showed an upregulation of *E2F3a* in tumor samples and our current results support that the other two E2F activator family members, *E2F1* and *E2F2*, are also over-expressed in tumor samples compared with the corresponding normal tissue. These data confirm those found *in vivo* using a mouse model, called TKO (Triple Knock Out): when Retinoblastoma family was conditionally deleted in bladder, E2F transcription factors increased their expression what led to the development of non-invasive muscle tumours, molecularly very similar to human NMIBC^4^.

In the case of the increased expression found for *cMYC*, its up-regulation has been widely described in several tumor types, intimately linked to critical processes in cancer such as proliferation, apoptosis, or metastasis^22,23^. In the case of BC, it has been related to the development of gemcitabine chemo-resistance^24^. Interestingly, several studies have pointed towards a narrow link between MYC expression and the correct work of splicing machinery^25,26^, so it could be very interesting to further study this relationship in the case of BC.

But transcriptome analyses not only showed the involvement of cMYC and E2F, the deregulated genes showed also that processes such as RNA splicing, ncRNA processing, and RNA processing were involved in tumour initiation. To further unravel the role of splicing, we studied which genes correlated with the expression of three different splicing factors, finding a high correlation with epigenetic modifiers (*JARID1A*, *HOXC9*) and again c-*MYC* and *E2F1*. Growing evidences have proved that AS events are involved in oncogenic processes such as proliferation, apoptosis, hypoxia, angiogenesis, immune escape and metastasis^7,27–29^. In addition, oncogenic isoforms generated by AS have been associated with clinical outcome and cancer risk in several tumor types^9,30,31^. In BC, particular AS events have been described associated to worse prognosis^11,32^. Bielli *et al*. have recently described that the expression of the splicing factor *PTBP1* correlates with disease progression, poor prognosis and worse survival in NMIBC patients^10^. However, research has been generally focused on particular events and comprehensive analyses are scarce. Last year, He *et al*. described prognostic signatures using splicing events to predict the prognosis of MIBC patients. Here, for the first time, we focus on the differential exon usage in earlier stages (NMIBC). Exons showed statistically differences in their expression (or usage) between normal and tumor samples. These genes belonged to fundamental bladder cancer signalling pathways such as Notch and Hedgehog. By studying in detail the expression of their corresponding read-out genes to try to understand the global effect on pathway activity caused by splicing regulation, we found a decreased expression of *HES/HEY* family in tumor samples, suggesting that AS would help to the down-regulation of Notch pathway. These data support the Maraver and cols’ results^17^ that demonstrated that bladder tumours with low levels of *HES1* promoted more mesenchymal and invasive features, favouring tumor progression and, therefore, a worse prognosis. In the case of Hedgehog signalling, higher tumor expression was detected for the read-out genes *GLI1* and *GLI2*. In this case, AS would support the pathway activation, helping consequently to the tumor development and spread as previously demonstrated in BC^18,33^. Although particular “tumor-specific” isoforms are still to be further determined, it seems clear that the new generated isoforms helps tumor to grow and expand.

In the case of the differences in AS between patients’ tumours developing, or not, recurrence, the differences showed less statistical power than in the previous comparison normal versus tumor, probably due to fewer differences among tumour samples compared to tumour vs normal, and also to the lower number of comparisons (28 vs 38). Still, GOBP analyses showed an involvement of classical cancer-key genes (*SMAD4*, *STAT3*, *RUNX1*, *TP53*,…), and again epigenetic factors (*JARID1A*, *KDM5B*), *E2Fs*, and *MYC*, as in the previous normal-tumor comparison. The involvement again of E2F family and cMYC indicates their important role not only for the tumor initiation but also in the recurrence. In a previous work^4^, we already described that those patients with higher *E2F3a* expression suffered more and earlier recurrences, although the same relationship is not found for *E2F1* and *E2F2*. This can be explained by the highly significant and positive correlation between *E2F3a* and EZH2, the core of the epigenetic Polycomb group expression, that mediates bladder tumor recurrence^4^. These new results demonstrate that the epigenetic regulation is not only restricted to Polycomb complex, but also to other factors such as *JARID1A* and *KDM5B*. In fact, alterations in other family members and epigenetic factors, as *ARID1A*, *UTX* or *TERT*, have been already proposed as bad prognosis factors in BC^34–37^.

Among the genes with AS related to recurrence, it attracted our attention the gene *MDM2*, repressor of TP53. It showed clear differences in several exons between recurrent and non-recurrent samples. Our results showed that around first and last exons are more expressed in samples from patients suffering recurrences. This could indicate that recurrence can be favoured by shorter isoforms that span intermediate exons, keeping uniquely the first and last exons. These shorter isoforms have been previously described as faster transcribed and unable to interact with TP53 in other tumours^38,39^. To test this hypothesis and taken into account that deregulated genes showed binding sites to TP53, we decided to study TP53 protein expression. An increased protein expression in samples from those patients suffering tumor recurrence was detected, supporting that the shorter isoforms were not binding TP53 and somehow favouring recurrence. In order to check if this TP53 high expression can be helped by the mutational stage, we used NGS. We found a close association between mutated *TP53* and more advanced stage and grade, similar to that already reported already in the case of MIBC^14,15^. However, no association between genomic variants and recurrence was found. Although our patient cohort includes few cases of *exitus*, our data showed that those patients with mutations in *TP53* present a lower disease specific survival. Therefore, according our data, mutations in TP53 are more related to tumor progression and prognosis, while its protein expression does correlate with recurrence. These results are in agreement with those from Pietzak *et al*. that reported frequent alterations in TP53 in their NMIBC samples (21%), being more frequently altered in advanced stages and grades. However, when they examined recurrence with TP53 altered-tumours, alone and combined with MDM2 after BCG treatment, no association was detected^40^. Interestingly, Zhou *et al*. did find an association between overexpression in NMIBC treated with BCG and recurrence free survival^41^. However, this association has not been always determined. For example, Vetterlein *et al*. did not detect association between TP53 positivity and disease progression, although in this case only patients with pT1 tumours were considered^42^. Finally, Hedegaard *et al*.^19^ recently published the most comprehensive transcriptome analysis of NMIBC samples, supporting a higher TP53 expression in more advanced stages, and more frequent mutations in high-risk patients, according with our current results. Recently, it has been published that MDM2 can bind EZH2 in a independent TP53 manner^43^, so it would be really interesting to explore this new path in NMIBC. Curiously, when focused on a population variant for codon 72, the heterozygous patients showed a statistically higher probability of cancer recurrence. The status of this codon of *TP53* has been linked to cancer progression in different tumor types^44–46^, although its value in BC needs further research in a wider dataset.

As a whole, our gene expression data indicate that an up-regulation of *E2F* family and *MYC* participates in both initiation and tumor progression through recurrence, regulated by epigenetic mechanisms. RNA processing mechanisms are crucial for bladder tumor initiation and AS events affect key pathways to favour bladder cancer development. Finally, an alternative splicing of *MDM2* could be favouring a higher TP53 protein expression and, as a consequence, favouring the recurrence development. In addition, mutations in *TP53* are related to advanced stage, grade, and worse prognosis. Therefore, these results open a new possibility to use the status of TP53 as a prognosis biomarker in NMIBC.

## Methods

### Patients

Informed consent was obtained from all patients. The Ethical Committee for Clinical Research of “University Hospital 12 de Octubre” approved the study. Samples and clinical united data from patients were provided by the Biobanco i+12 in the Hospital 12 de Octubre integrated in the Spanish Hospital Biobanks Network (RetBioH; www.redbiobancos.es), following standard operation procedures with appropriate approval of the Ethical and Scientific Committees. Their baseline characteristics are included in Table 1 and the sampling workflow is summarized in Suplementary Fig. 1A. The patients have been consecutively evaluated at the Urology Department of the University Hospital “12 de Octubre” and diagnosed with BC (Ta-T1-T2), following current European Guidelines. All experiments and methods were performed in accordance with relevant guidelines and regulations. The sample recollection and preservation procedures have been reported elsewhere^3,4,47^.

**Table 1:** Baseline characteristics of patients.

Patients (n)	82*
Age median (range)	72.4 years (49–89)
Sex (M: male; F: female)	M = 63
	F = 19
Smoker status	No = 15
	Currently smoker = 27
	Ex smoker = 38
	ND = 2
Stage	Papilloma = 1
	Ta = 36
	T1 = 35
	T2 = 10
Grade	Papilloma = 1
	Low = 43
	High = 33
	PUNLMP = 4
	ND = 1
Alterations in normal mucosa	Dysplasia = 6
	Metaplasia = 1
	Glandular cystitis = 1

ND: no data.

*This is the total number of patients included in the study. The number of tumor samples varies in each analyses depending on the required material available. See Supplementary Fig. 1.

### Whole transcriptome analyses and RT-qPCR

Total RNA was isolated using miRNeasy Mini Kit (Qiagen) according to the manufacturer’s instructions and DNA was eliminated (RNAse-Free DNAse Set Qiagen). Genome-wide transcriptome experiments were performed using the Affymetrix HuGene-1_0-st-v1 microarray at the Genomics Facility of the Cancer Research Center (Salamanca, Spain) using standard procedures as previously reported^4^. Datasets have been deposited in GEO (GSE38264). Gene Ontology and Chip Enrichment Analysis were performed using Enrich webtool (http://amp.pharm.mssm.edu/Enrichr/). Gene Set Enrichment Analysis (GSEA) was performed using the MSignature and Motif databases. To analyse relative gene expression patterns, PTM (Pavlidis Template Matching) test was used. RNA-Seq data from the most extensive and comprehensive transcriptome analysis for NMIBC were also analysed, where 476 samples are included^19^.

For RT-qPCR analyses, reverse transcription was performed using the Omniscript RT Kit (Qiagen) and a primer specific for each of all genes of interest using 10 ng of total RNA. The sequences of the specific oligonucleotides used are listed in Table 2. qPCR was carried out in a 7500 Fast Real Time PCR System using Go Taq PCR master mix (Promega) and 1 µL of cDNA as a template. Melting curves were performed to verify specificity and absence of primer dimerization. Reaction efficiency was calculated for each primer combination, and *TBP* gene was used as reference gene for normalization^48^.

**Table 2:** Oligo sequences for RT-qPCR analyses from 5′ to 3′.

Name (RT: specific for retrotranscription F: forward R: reverse)	Sequence (5′-3′)
E2F1_RT	GTA TAA ATT AAA TGT TTC CA
E2F1_F	ACT CAG CCT GGA GCA AGA AC
E2F1_R	GAG AAG TCC TCC CGC ACA T
E2F2_RT	GAA GTG TCA TAC CGA GTC TTC TCC
E2F2_F	TCC CAA TCC CCT CCA GAT C
E2F2_R	CAA GTT GTG CGA TGC CTG C
MYC-RT	GTT AGA AGG AAT CG
MYC-F	AAT GAA AAG GCC CCC AAG GTA GTT ATCC
MYC-R	GTC GTT TCC GCA ACA AGT CCT CTT C
HES1_RT	GTG CGC ACC TCG GTA TTA AC
HES1_F	GAA GCA CCT CCG GAA CCT
HES1_R	GTC ACC TCG TTC ATG CAC TC
HEYL_RT	GGG CAT CAA AGA ATC CTG TC
HEYL_F	GTC CCC ACT GCC TTT GAG
HEYL_R	ACC GTC ATC TGC AAG ACC TC
HEY1_RT	AGC AGA TCC CTG CTT CTC AA
HEY1_F	CGA GCT GGA CGA GAC CAT
HEY1_R	GGA ACC TAG AGC CGA ACT CA
GLI1_RT	TGACTTCTGTCCCCACACTG
GLI1_F	AGCGCCCAGACAGAGTGT
GLI1_R	GGGGTCATCGAGTTGAACAT
GLI2_RT	AGCTGGCTCAGCATGGTC
GLI2_F	ACTCCACACACGCGGAAC
GLI2_R	CCACTGAAGTTTTCCAGGATG
PTCH1_RT	CGA GGT TCG CTG CTT TTA AT
PTCH1_F	TCT GGA GCA GAT TTC CAA GG
PTCH1_R	TTT GAA TGT AAC AAC CCA GTT TAA ATA
TBP_RT	GTG TTT AAA ATC TAC ATA
TBP_F	AGT GAA GAA CAG TCC AGA CTG
TBP_R	CCA GGA AAT AAC TCT GGC TCA T

### Exon array data analysis

Affymetrix HuGene-1_0-st-v1 microarray probe signals were summarised into probeset signals to provide a measure of expression of individual exons and observe differential exon inclusion or skipping using Bioconductor library “oligo”. Array quality evaluation was performed using Bioconductor library “arrayQualityMetrics”. RMA (Robust Multi-array Analysis) algorithm was used for the normalization and summarization of the data. Three different filters were then applied: (1) to eliminate those probes with a ‘crosshyb_type’ different of 1 (probes that hybridise to sequences other than the target sequence). (2) Following the Affymetrix technical note^49^, a probeset could be considered detected when the DABG (‘Detected above background’) *p* < 0.05 in ~50% of the samples of at least one group. Based on this, APT command was used to apply this filter following to Lockstone^50^. (3) Those exons unique for a transcript were eliminated. Once data were filtered, Splicing Index (SI) was calculated as the log2 ratio of exon to gene intensity. Finally, statistically differences were detected using the “Limma” R package from Bioconductor. Multiple testing correction was applied using the Bonferroni adjustment.

### Tissue microarray (TMA) and Immunohistochemistry

The construction and analysis of tissue microarray containing all these human samples has been reported elsewhere^4,47^. At least two representative duplicate cores for each case were scored. Inmunohistochemistry analysis was carried out using the monoclonal mouse anti-human p53 (DO07, Agilent-Dako). Signal was amplified using avidin-peroxidase (ABC Elite Kit; Vector Labs), and peroxidase was visualized using 3,30-diaminobenzidine as a substrate (DAB kit, Vector Labs). Negative control slides were obtained by replacing primary antibodies with PBS (data not shown). Scoring of the results and selection of the thresholds, internal controls for reactivity of each antibody, and tissue controls for the series were done according to previously published methods^4,47^.

### Genomic TP53 analysis

DNA was obtained using DNeasy Blood & Tissue Kit from Qiagen following manufacturer’s instructions. In order to identify single nucleotide variant (SNVs) and small indels in the coding region of *TP53* (excluding promoter 5′ or 3′ UTR), the TP53 MASTR molecular assay from Multiplicon (Agilent) was used. The pipeline for SNV calling consisted on the mapping of the raw reads using the software package BWA followed by SNV calling using SAMtools mpileup command. The following quality-filter criteria were applied to VCF files: all variants should present a value in the QUAL field >= 20, total DP4 >= 20, and definitive genotype GT:PL >= 100. Finally, Ensembl Variant Effect Predictor (VEP, release 75) was used for the variant annotations.

### Statistical analysis

Comparisons between normal and its corresponding tumor samples were studied by paired sample T-test. Contingency analyses were made using the Fisher exact test. Recurrence and disease free survival analyses were performed using the Kaplan–Meier method and statistical differences between the patient groups were tested by the log-rank test. For RNA-Seq analyses from Hedegaard *et al*.^19^, FPKM (Fragments Per Kilobase Million) data was compared between clinical characteristics using T-test analyses, according a previous Levene test. SPSS 17.0 and GraphPad Prism 6.0 software were used. For the differential exon usage analysis, Limma and Bonferroni multiple testing correction were done in R.

## Supplementary information

Supplementary Figure 1: Flowchart of number of patients and RT-qPCR analyses
