# Supplementary material for: Gene Expression Analyses in Non Muscle Invasive Bladder Cancer Reveals a Role for Alternative Splicing and Tp53 Status
Source: Sci Rep. 2019 Jul 17;9:10362. doi: 10.1038/s41598-019-46652-4 (PMC6637137; doi:10.1038/s41598-019-46652-4)
Supplement: Supplementary file 1 — Supplementary Figure 1: Flowchart of number of patients and RT-qPCR analyses [file 41598_2019_46652_MOESM1_ESM.pdf]

Supplementary Figure 1: Flowchart of number of patients and RT-qPCR analyses

Gene expression analyses in non muscle invasive bladder cancer reveals a role for alternative splicing and TP53 status

Marta Dueñas, Andrés Pérez-Figueroa, Carla Oliveira, Cristian Suárez-Cabrera, Abel Sousa, Patricia Oliveira, Felipe Villacampa, Jesús M Paramio, Mónica Martínez-Fernández

Supplementary Figure 1. A) Flowchart of the patient number used for each purpose. **B)** RT-qPCR results measuring relative gene expression using TBP as housekeeping gene for E2F1, E2F2 and cMYC. The three genes showed higher expression in tumor samples compared with their paired normal samples.

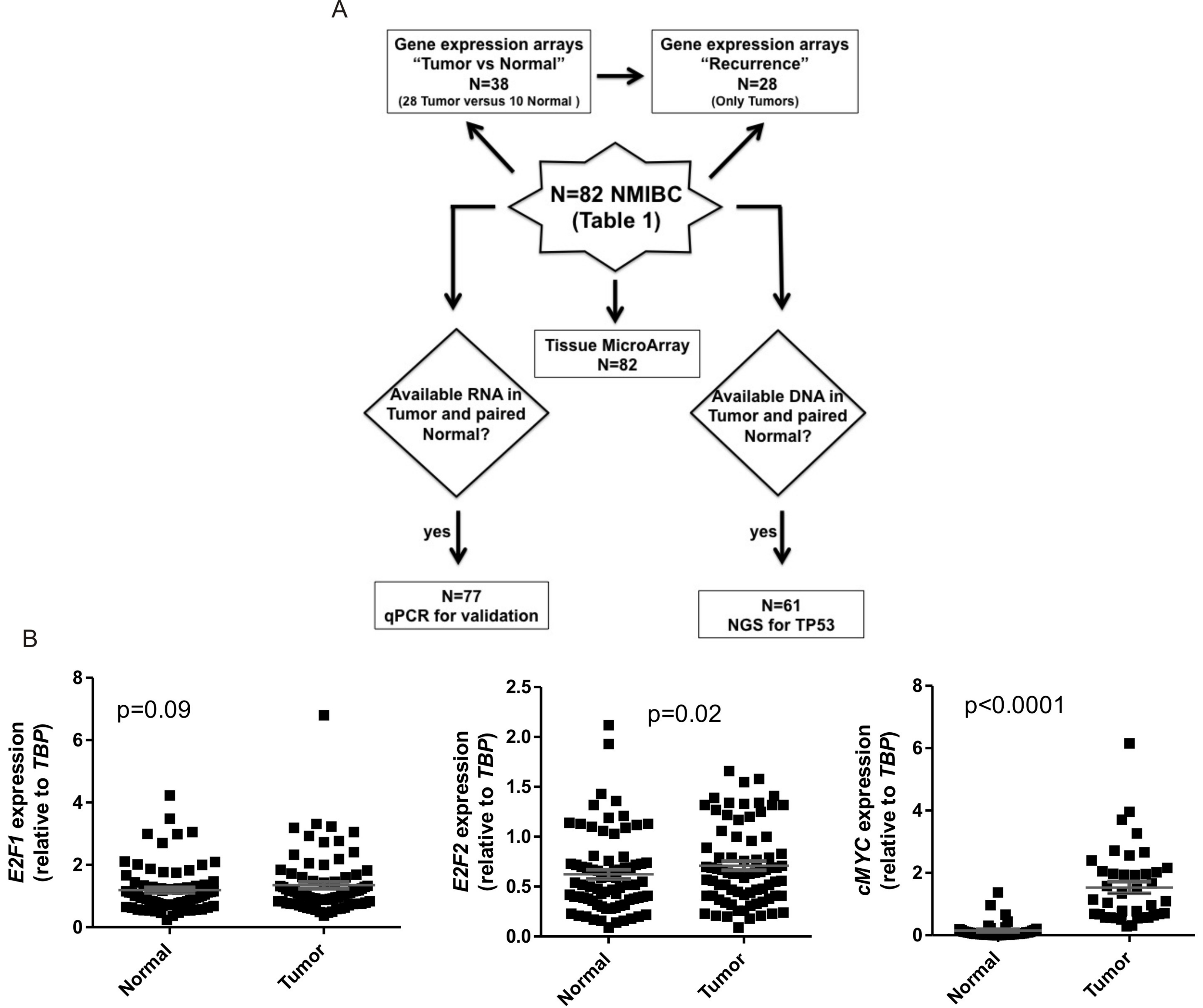

A
Gene expression arrays
"Tumor vs Normal"
N=38
(28 Tumor versus 10 Normal)
Gene expression arrays
"Recurrence"
N=28
(Only Tumors)
N=82 NMIBC
(Table 1)
Tissue MicroArray
N=82
Available RNA in
Tumor and paired
Normal?
Available DNA in
Tumor and paired
Normal?
yes
yes
N=77
qPCR for validation
N=61
NGS for TP53
B
E2F1 expression
(relative to TBP)
p=0.09
Normal
Tumor
E2F2 expression
(relative to TBP)
p=0.02
cMYC expression
(relative to TBP)
p<0.0001
